# Seasonality in depressive and anxiety symptoms among primary care patients and in patients with depressive and anxiety disorders; results from the Netherlands Study of Depression and Anxiety

**DOI:** 10.1186/1471-244X-11-198

**Published:** 2011-12-19

**Authors:** Wim H Winthorst, Wendy J Post, Ybe Meesters, Brenda WHJ Penninx, Willem A Nolen

**Affiliations:** 1 Department of Psychiatry, University Medical Centre Groningen, University of Groningen, the Netherlands; 2 Department of Epidemiology, University Medical Centre Groningen, University of Groningen, the Netherlands; 3 Department of Pedagogy & Educational Sciences, University of Groningen, the Netherlands; 4 Department of Psychiatry/EMGO Institute/Neuroscience Campus Amsterdam, VU University Medical Center, Amsterdam, the Netherlands; 5 Department of Psychiatry, Leiden University Medical Centre, Leiden, the Netherlands

## Abstract

**Background:**

Little is known about seasonality of specific depressive symptoms and anxiety symptoms in different patient populations. This study aims to assess seasonal variation of depressive and anxiety symptoms in a primary care population and across participants who were classified in diagnostic groups 1) healthy controls 2) patients with a major depressive disorder, 3) patients with any anxiety disorder and 4) patients with a major depression and any anxiety disorder.

**Methods:**

Data were used from the Netherlands Study of Depression and Anxiety (NESDA). First, in 5549 patients from the NESDA primary care recruitment population the Kessler-10 screening questionnaire was used and data were analyzed across season in a multilevel linear model. Second, in 1090 subjects classified into four groups according to psychiatric status according to the Composite International Diagnostic Interview, overall depressive symptoms and atypical versus melancholic features were assessed with the Inventory of Depressive Symptoms. Anxiety and fear were assessed with the Beck Anxiety Inventory and the Fear questionnaire. Symptom levels across season were analyzed in a linear regression model.

**Results:**

In the primary care population the severity of depressive and anxiety symptoms did not show a seasonal pattern. In the diagnostic groups healthy controls and patients with any anxiety disorder, but not patients with a major depressive disorder, showed a small rise in depressive symptoms in winter. Atypical and melancholic symptoms were both elevated in winter. No seasonal pattern for anxiety symptoms was found. There was a small gender related seasonal effect for fear symptoms.

**Conclusions:**

Seasonal differences in severity or type of depressive and anxiety symptoms, as measured with a general screening instrument and symptom questionnaires, were absent or small in effect size in a primary care population and in patient populations with a major depressive disorder and anxiety disorders.

## Background

Epidemiological studies of seasonal variation in the prevalence of mental disorders have shown diverging results. Seasonal variation in the prevalence of the major mental disorders in general population surveys have rarely been noted, but prevalence rates of mood (affective) disorders with a seasonal pattern have been reported to range from 1% to as much as 12% [1].

The majority of the latter studies reported on seasonal affective disorder (SAD), defined in DSM IV as a recurrent depressive disorder with a regular temporal relationship between the onset of a major depressive episode and a particular time of the year (mostly fall or winter) and has used specific instruments for its assessment [2,3]. The most widely used instrument in those studies is the Seasonal Pattern Assessment Questionnaire (SPAQ), a self rating screening questionnaire that retrospectively measures seasonal variation in mood, social activities and atypical depressive symptoms such as increased sleep, increased appetite and weight and a lowered energy level [4]. Female gender and young age have been described to be associated with a higher prevalence of SAD [5,6]. The influence of latitude on the prevalence of SAD has been suggested but could not be demonstrated [7,8].

Absence of seasonal variation in the prevalence of mental disorders has been reported in studies in which data were collected using general structured interviews or questionnaires on depression in different months of the year. For example, in New England (USA) in a study involving 1,500 patients of a psychiatric outpatient practice using the Structured Clinical Interview for DSM-IV (SCID), there were no higher rates of onset of major depressive disorders in spring and fall, and no higher rates of atypical depression in the winter compared to the other seasons [9]. In a multicenter study on the current prevalence of depression in the United Kingdom, Finland, Norway and Spain among 6608 participants randomly identified via census registers or primary care databases and using the Beck Depression Inventory (BDI), also no evidence of a systematic seasonal pattern in depression was found [10]. In Iceland no seasonal mood change could be demonstrated in a cross sectional study using the Hospital Anxiety and Depression Questionnaire in four 1000-person cohorts who received the questionnaire in either January, April, July or October [11]. Similarly, in a Dutch general population survey among 7076 participants (NEMESIS), and using the Composite International Diagnostic Interview (CIDI), no seasonal difference was found in the 1-month prevalence of the main categories of mood disorders or for the broad category of anxiety disorders [12]. And finally, in a UK study among 2,255 patients consulting their general practitioner who were screened over the course of a year using the General Health Questionnaire (GHQ 30), no significant seasonal variation in GHQ scores was found [13].

However, other studies also using general structured interviews or questionnaires to assess depression did report seasonal variations. In another Dutch general population study among 5356 participants, a higher mean score on the Centre for Epidemiological Studies Depression Scale (CES-D) was found in the winter compared to the summer [14]. In a general population study in Norway among 11054 participants, modest variations in the Hospital Anxiety and Depression Scale (HADS) were found, mean sum scores being slightly higher during November through March compared to the other months [15]. In a US study among 1556 men and 314 women using the Hopkins Symptom Checklist, women scored significantly higher in winter on the expanded mood scale [16]. Finally, in the US National Comorbidity Survey among 8,089 participants and using CIDI, a lifetime prevalence of major depression with a seasonal pattern of 0.4% was found, and a prevalence of major or minor depression with a seasonal pattern of 1.0%[17].

In addition, the studies mentioned above did not measure seasonality in severity of atypical depressive symptoms, melancholic depressive symptoms and anxiety symptoms in specific patient groups with depressive and anxiety disorders.

The aim of this study was to determine if seasonal variation exists in the severity and type of depressive and anxiety symptoms in general and among patients with a depressive or anxiety disorder. More specific three questions were formulated: (1) Does a seasonal pattern exist in the severity of depressive and anxiety symptoms among patients visiting their general practitioner for any reason?

(2) Does a seasonal pattern exist in the severity of depressive or anxiety symptoms among patients with a current depressive disorder, a current anxiety disorder, a current depressive and anxiety disorder, and among healthy controls; and is there a difference between these groups?

(3) Does a seasonal pattern exist in type of depressive symptoms (i.e. atypical or melancholic) among and between these groups?

## Methods

The study was conducted using data from the Netherlands Study of Depression and Anxiety (NESDA, http://www.nesda.nl): (1) the NESDA primary care recruitment population and (2&3) the NESDA baseline population [18]. NESDA is an ongoing multi-site naturalistic 8-year longitudinal cohort study among 2,981 adults (18-65 years), aimed at describing the long-term course and consequences of depressive and anxiety disorders. The NESDA sample (total n = 2981) is stratified for setting: community, primary care and specialized mental health care. The community sample (n = 564) was built on two cohorts that were already available through prior studies described in detail elsewhere [19]. The primary care participants (n = 1610) were recruited among 23,750 patients from practices of 65 general practitioners (GPs) in the vicinity of three research sites. The specialized mental health patients (n = 807) were recruited from outpatient clinics of regional facilities for mental health care around the research sites.

Across recruitment settings, uniform inclusion and exclusion criteria were used. The NESDA sample included a range of psychopathology: those with no lifetime anxiety or depressive disorders (including healthy controls), those with a current first or recurrent depressive disorder (major depressive disorder or dysthymic disorder) or anxiety disorder (panic disorder with or without agoraphobia, agoraphobia, social phobia or generalized anxiety disorders) and those with earlier episodes, or at risk because of sub threshold symptoms or a positive parental history for depressive or anxiety disorders. Excluded were patients with a psychotic disorder, bipolar disorder, obsessive compulsive disorder, or severe substance use disorder, and persons not fluent in Dutch.

### Ethics Statement

The study protocol of NESDA was approved by the Ethical Review Board of the VU University Medical Center, the Leiden University Medical Center and the University Medical Center Groningen. After full verbal and written information about the study, written informed consent was obtained from all participants at the start of baseline assessment. A full ethics statement of NESDA and detailed information on objectives and methods of NESDA were published elsewhere [18].

### Subjects

The NESDA primary care recruitment population, to whom the Kessler-10 screening questionnaire was sent, consisted of a random sample of all patients who had visited their GP during the previous four months for any reason from January 2004 to February 2007. The date the questionnaire was filled out was recorded for participants from the research sites in Amsterdam and Groningen (latitude 52,3° and 53,2° respectively). Questionnaires with two or more answers missing were excluded.

The NESDA baseline population comprised participants of the NESDA cohort who met the criteria of one of four groups: 1) Healthy controls (HC), i.e. no lifetime depressive or anxiety disorder; 2) Major depressive disorder (MDD) last month; 3) Any anxiety disorder (AAD) last month; 4) Both major depressive disorder and any anxiety disorder (MDD + AAD) last month. Participants with a lifetime MDD or AAD but not within the last month, and those not completing the self report questionnaires (see below) within 7 days of the baseline-interview, were left out of the analysis.

### Measures

In the NESDA primary care recruitment population (n = 5,549) the Kessler-10 screening questionnaire (K-10) was used. The K10 has proven screening qualities for affective disorders based on questions about anxiety and depressive symptoms that a person has experienced in the past 4 weeks [20,21].

In the NESDA baseline population (n = 1,090) the CIDI (WHO version 2.1) was used to establish diagnoses of MDD and AAD according to DSM-IV criteria (American Psychiatric Association, 2001). Within 7 days prior or after the CIDI interview, all participants completed several self-report questionnaires. Severity of depression was assessed with the Inventory of Depressive Symptoms, 30 item self-report versions (IDS) [22]. Moreover, the IDS was used to assess the presence and severity of atypical and melancholic features, as the IDS includes all symptoms of these specifiers. Therefore a continuous atypical specifier was constructed (At-IDS): a summation of the scores on the items mood reactivity, the highest score of either weight gain or increase in appetite, hypersomnia, leaden paralysis, and interpersonal rejection sensitivity (score range 0 - 3, total score range 0 - 15). The scores of the item mood reactivity were recoded (reversed) resulting in an item that counts the presence of the symptom mood reactivity in stead of its absence. Participants with one or more missing items were excluded from the analysis. Also a continuous melancholic specifier was constructed (Mel-IDS): a summation of the scores on the items: loss of pleasure, lack of reactivity to usually pleasurable stimuli, depressed mood, regularly worse in the morning, early morning awakening, psychomotor retardation or agitation, the highest score of either anorexia or weight loss, and excessive or inappropriate guilt (score range 0 - 3, total score range 0 - 24). Also for Mel-IDS participants with missing items were excluded from the analysis.

The Beck Anxiety Inventory (BAI), a 21-item self-report instrument, was used to assess overall severity of anxiety [23]. Finally the 15-item self-report version of the Fear Questionnaire (FQ) was used to measure severity of fear and avoidance [24].

### Statistical analysis

The dates were categorized into the four seasons (spring: March 21 - June 20, summer: June 21 - September 20, autumn: September 21-December 20, winter: December 21 - March 20). SPSS (SPSS 16.02 inc., 2008) and MLwin (2.02) were used to analyze the data. Descriptive analyses with means and standard errors for quantitative data were calculated. 95% Confidence intervals were calculated and a p-value smaller than 0.05 (two-sided) was considered to be significant.

### Question 1. Seasonality in severity of depressive and anxiety symptoms among primary care patients (recruitment population)

As the distribution of the K-10 total score was skewed and the assumption of normality was violated, the log transformed K-10 score (LnK10) was calculated and used as outcome variable. Taking into account the fact that each GP had several participants, and assuming that there could be dependency between participants within the practices of the GPs, multilevel analysis (by MLwin) was used to analyze the course over time. In this analysis the GP's were considered to be on the highest level, and participants on the lowest level. For the quantitative outcome measure (LnK10), a linear model was specified.

Analysis started with the empty model, a model only including an intercept with random terms. In this model, the different sources of variability (within GP's and between GP's) were distinguished. Then, different models for the time course were specified, based on the four seasons, and different combinations of fixed and random effects. Differences in deviance determined whether the different specifications of the time course were significant or not. Additionally, the predictors gender, age, and the location of the field site were included as fixed effects. Interaction terms were explored as well.

### Question 2 & 3. Seasonality in severity of depressive and anxiety symptoms and type of depressive symptoms in patients with a current depressive and/or anxiety disorder and in healthy controls

For all continuous outcome measures (IDS, At-IDS, Mel-IDS, BAI and FQ), a linear regression model was specified with group, season, age and gender as independent variables. Only significant main effects were included in the model. Analysis started with a model only including the four groups of participants. Then, different models were specified with the four seasons, age and gender as predictors. Based on literature and descriptive statistics, two way interactions between season, gender, age and group were analyzed. Significant interactions were additionally included in the model. Standardized regression coefficients were calculated and were used as a measure for the clinical relevance of the findings.

## Results

### Question 1. Seasonality in severity of depressive and anxiety symptoms among primary care patients (recruitment population)

A total of 23,750 questionnaires was sent out. 10,706 K-10 questionnaires were returned (45%). Those returning the K-10 (10,706) were more likely to be women (59.3% versus 50.0%, p < .001) and older (44.4 versus 39.0 years, p < .001) compared to those not returning the screener. The date the K-10 was filled out could be recovered for 5,563 participants from the field sites in Amsterdam and Groningen. Because these dates were not recorded in Leiden the participants from the field site Leiden were excluded from the analysis. Off the remainder 14 K-10 questionnaires had 2 or more answers missing; and were excluded as a consequence. The resuming 5,549 participants from 44 GPs were included in the analysis, consisting of 3664 (66%) women and 1885 (34%) men. The mean age was 43.6 years (SE = 0.17).

**Figure 1:**
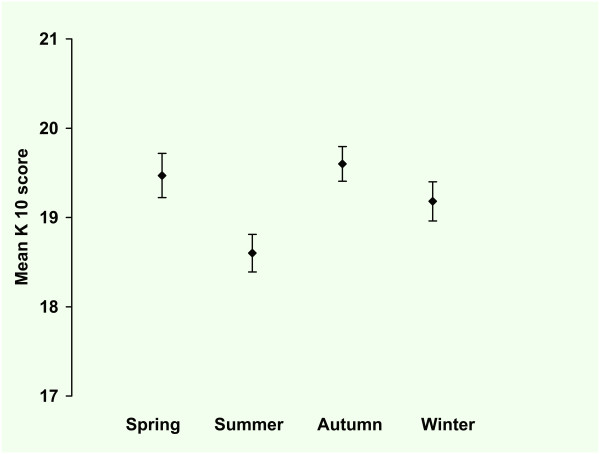
**Kessler-10 screening Questionnaire: mean score per season**. Primary care patients (n = 5549). Values are mean scores. Error bars represent standard errors of the mean. Seasons: spring (March 21 - June 20), autumn (September 21-December 20), winter (December 21 - March 20). There was no statistical difference between the seasons (defined as p < 0,05).

In Figure 1 the observed means and standard errors of the K-10 score are presented per season. The observed total mean K-10 score was 19.2 (SE = 0.11), the median score was 17 (range 10-50), the lowest scores were recorded in summer and the highest scores in autumn. The mean score for women was higher than the mean score for men. Older participants scored lower than younger participants, with younger women scoring higher than younger men. Amsterdam participants (n = 3392) scored higher than Groningen participants (n = 2157).

In table 1 the results of the multilevel regression analysis are presented for the log transformed K-10 scores. The second model with the seasons as a predictor (with spring as a reference), explains only little more variability than the empty model as can be seen in the difference of the deviance (empty model 5086.6; model with seasons 5085.6). In this second model the difference between de seasons was not significant (summer -0.014, SE 0.019; autumn -0.002, SE 0.021; winter -0.013, SE 0.021).

**Table 1:** Model of the log transformated scores of the Kessler-10 questionnaire

	Empty model^1^	Seasons^2^	Full model^3^
**Fixed Effect**	**β (SE)**	**β (SE)**	**β (SE)**

Intercept	2.887 (0.013)	2.863 (0.024)	2.984 (0.025)

Spring (reference)			

Summer		-0.014 (0.019)	-0.015 (0.018)

Autumn		-0.002 (0.021)	0.022 (0.019)

Winter		-0.013 (0.021)	-0.002 (0.019)

Men (reference)			

Women			0.065 (0.011)*

Age			-0.002 (0.000)*

Amsterdam (reference)			

Groningen			-0.127 (0.019)*

**Random Effect**			

Level two: General practitioner Intercept variance	0.007 (0.002)	0.007 (0.002)	0.002 (0.001)*

Level one: Individual variance	0.144 (0.003)	0.144 (0.003)	0.142 (0.003)

Deviance	5086.6	5085.6	4928.3

β = Beta

SE = standard error

* p < 0.05

^1) ^Empty model

^2) ^Model with seasons

^3) ^Full model with seasons and covariates

Adding the covariates gender, age and site the final model showed that these variables contribute significantly to the explanation of the model (gender 0.065, SE 0.011; age 0.002, SE 0.000; site -0.127, SE 0.019) but there was no significant difference between the seasons (summer -0.015, SE 0.018; autumn -0.022, SE 0.019; winter -0.002, SE 0.019). No significant interactions were found between the seasons and these covariates, nor between the covariates themselves.

Back transformation of the log transformed K-10 scores revealed that women scored 1.07 higher than men and participants in Amsterdam scored 1.15 higher than participants in Groningen. On the highest level, there was a significant difference of 1.01 points between the GP's.

### Question 2 & 3. Seasonality in severity of depressive and anxiety symptoms and type of depressive symptoms in patients with a current depressive and/or anxiety disorder and in healthy controls

Data comprised 1,090 participants (691 women = 63.4%) of the NESDA cohort (2,981 participants) who met the criteria of one of four groups and completed the IDS: HC (n = 465), MDD (n = 131), AAD (n = 134), MDD + AAD (n = 360). The BAI and the FQ had one participant missing, resulting in 1089 included participants. 16 Participants were excluded due to missing items on At-IDS (1.5%) resulting in 1074 participants in the analysis of At-IDS. 57 Participants were excluded due to missing items on Me-IDS (5.2%) resulting in 1033 participants in the analysis of Me-IDS.

### 2.1. Severity of depressive symptoms

**Figure 2:**
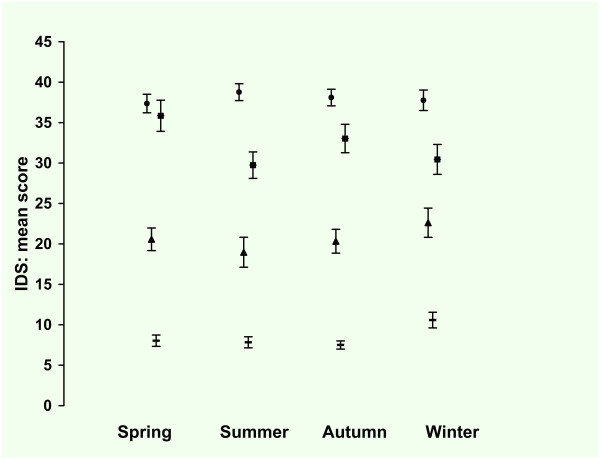
Inventory of Depressive Symptoms*: mean score per season. *30 Item self-report version of the Inventory of Depressive Symptoms. Total group (n = 1090), ▬ = Healthy Control (n = 465), ▲ = Any Anxiety disorder last month (n = 134), ■ = Major Depression last month (n = 131), ● = Major Depression and Any Anxiety Disorder last month (n = 360). Values are mean scores. Error bars represent Standard Errors of the mean.

Figure 2 presents the observed means and standard errors of the IDS by season for the four groups. The observed mean score was lowest for autumn (20.9, SE 0.90) and highest for winter (25.7, SE 1.00), with intermediate scores for spring (22.0, SE 1.01) and summer (21.7, SE 1.01). As expected, the observed mean score increased with the severity of the pathology: HC scored 8.2 (SE 0.34), patients with AAD 20.7 (SE 0.83), patients with MDD 32.1 (SE 0.93) and patients with MDD + AAD 38.0 (SE 0.57). Taking all seasons together, the observed mean score for men was 21.2 (SE 0.83) and for women 23.2 (SE 0.61).

In tables 2 and 3 the results of the regression analysis are presented. In the model with only groups as predictor, the difference between the groups was significant with a medium to large effect size. In the second model with the seasons as predictor there were no significant differences between the seasons. Adding the predictors gender and age revealed that women scored significantly higher than men but there was no significant age effect. In this model with seasons and covariates there was no significant difference between the seasons. In the full model with seasons, covariates and interactions there were significant two way interactions between gender and season, meaning that the difference in score between men and women varied per season. Women scored higher than men in spring and summer (+1.0, +4.7 resp.), the difference diminishing in autumn (+ 0.2) and reversing in winter with women scoring lower than men (-0.6). There was also a significant two way interaction between group and season: the difference between winter and summer was 3.7 points smaller for MDD patients (+2.6) than for the other groups (+6.3). There was no significant main effect of age, nor were there any significant two way interactions between age and group, age and season, age and gender, and gender and group. In the final model the effect size was large for the groups but small for the seasons and interactions terms as can be seen from the unstandardized and standardized regression coefficients.

**Table 2:** IDS total score: regression model with groups and model with seasons

Main Effects	B	SE	LB	UB	β	p	B	SE	LB	UB	β	p
Intercept	8.24	0.43	7.40	9.08		< 0.01*	7.90	0.66	6.60	9.20		< 0.01*

HC (reference)												

MDD	23.86	0.92	22.06	25.66	.73	< 0.01*	23.74	0.92	21.94	25.55	.73	< 0.01*

AAD	12.45	0.91	10.67	14.23	.38	< 0.01*	12.34	0.91	10.55	14.14	.38	< 0.01*

MDD + AAD	-6.55	1.31	-9.12	-3.98	-.19	< 0.01*	-6.41	1.32	-8.99	-3.83	-.19	< 0.01*

Summer (reference)												

Autumn							0.14	0.77	-1.38	1.65	.00	0.86

Winter							1.09	0.82	-0.53	2.70	.03	0.19

Spring							0.46	0.82	- 1.16	2.07	.01	0.58

IDS = Inventory of Depressive Symptoms

B = Unstandardized Coefficient

SE = standard error of B

LB = Lower Bound of 95% Confidence Interval for B

UB = Upper Bound of 95% Confidence Interval for B

β = Standardized Coefficient

* p < 0.05

HC = Healthy Control

MDD = Major Depressive Disorder

AAD = Any Anxiety Disorder

MDD + AAD = Major Depressive Disorder + Any Anxiety Disorder

Note: adjusted R^2 ^Model with groups = 0,675

Note: adjusted R^2 ^Model with seasons = 0,674

**Table 3:** IDS total score: regression model with groups, seasons, covariates and full model with interactions

Main Effects	B	SE	LB	UB	β	p	B	SE	LB	UB	β	p
Intercept	5.83	1.18	3.50	8.15		< 0.01*	4.60	1.00	2.64	6.56		< 0.01*

HC (reference)												

MDD	23.75	0.92	21.95	25.55	.72	< 0.01*	24.80	0.97	22.90	26.69	.76	< 0.01*

AAD	12.37	0.91	10.58	14.16	.38	< 0.01*	12.36	0.91	10.58	14.15	.38	< 0.01*

MDD + AAD	-6.51	1.31	-9.09	-3.94	-.19	< 0.01*	-6.63	1.31	-9.19	-4.06	-.19	< 0.01*

Summer (reference)												

Autumn	0.12	0.77	-1.40	1.63	.00	0.88	2.94	1.25	0.48	5.39	.08	0.02*

Winter	1.08	0.82	-0.53	2.70	.03	0.19	6.28	1.50	3.35	9.21	.16	< 0.01*

Spring	0.45	0.82	-1.16	2.07	.01	0.59	2.72	1.37	0.04	5.40	.07	0.05*

Men (reference)												

Women	1.36	0.58	0.21	2.50	.40	0.002*	4.70	1.18	2.38	7.02	.14	< 0.01*

Age	0.03	0.02	-0.01	0.07	.02	0.16						

**Two way interactions**												

Winter & MDD							-3.74	1.33	-6.35	-1.13	.08	< 0.01*

Autumn & Women							-4.53	1.58	-7.64	-1.43	-.11	< 0.01*

Winter & Women							-5.34	1.68	-8.63	-2.04	-.12	< 0.01*

Spring & Women							-3.72	1.70	-7.07	-0.38	-.08	0.03*

IDS = Inventory of depressive Symptoms

B = Unstandardized Coefficient

SE = standard error of B

LB = Lower Bound of 95% Confidence Interval for B

UB = Upper Bound of 95% Confidence Interval for B

β = Standardized Coefficient

* p < 0.05

HC = Healthy Control

MDD = Major Depressive Disorder

AAD = Any Anxiety Disorder

MDD + AAD = Major Depressive Disorder + Any Anxiety Disorder

Note: adjusted R^2 ^Model with seasons and covariates = 0,675

Note: adjusted R^2 ^Full model with seasons, covariates and interactions = 0,680

### 2.2 Severity of anxiety symptoms (BAI)

**Figure 3:**
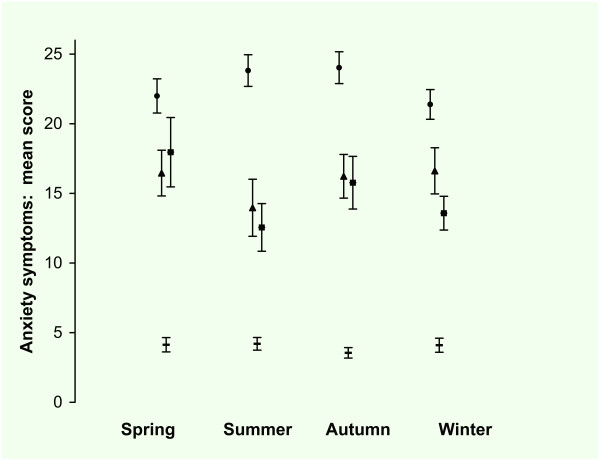
Becks Anxiety Inventory*: mean score per season. *21-item self-report version. Total group (n = 1089), ▬ = Healthy Control (n = 465), ▲ = Any Anxiety disorder last month (n = 133), ■ = Major Depression last month (n = 131), ● = Major Depression and Any Anxiety Disorder last month (n = 360). Values are mean scores. Error bars are Standard Errors of the mean.

The observed mean score was lowest for autumn (12.3 SE 0.69) and highest for winter (13.9 SE 0.70). The observed mean score for men was 11.9 (SE 0.59) and for women 13.6 (SE 0.46). The observed mean score for HC was 3.9 (SE 0.23), for patients with AAD 15.8 (SE 0.88), for patients with MDD 14.9 (SE 0.90) and for patients with MDD + AAD 13.0 (SE 0.36). In Figure 3 the observed means and standard errors of the BAI are presented by season for the four groups.

In tables 4 and 5 the results of the regression analysis are presented. In the model with only groups as predictor, the difference between the groups was significant with a medium to large effect size. In the second model with the seasons as predictor there were no significant differences between the seasons. Adding the predictors gender and age revealed that women scored significantly higher than men but there was no significant age effect. In this model with seasons and covariates there was still no significant difference between the seasons. In the full model with seasons, covariates and interactions there were significant two way interactions between season and group: Patients with a MDD scored lower in winter compared to summer (-2.9) and patients with MDD + AAD scored lower in spring compared to the summer (-2.9). There were significant main effects for the groups: patients with MDD and AAD scored higher than HC (+ 11.8). This was reduced in winter for patients with MDD (+ 8.9). Patients with MDD + AAD scored higher than HC (+20.2) which was reduced in winter (+ 17.3). There was a significant main effect for gender; women scored higher than men (+1.2). There was no significant main effect of age and there were no significant two way interactions between age and gender, age and season, age and group, gender and group or season and gender. In the final model the effect size was large for the groups but small for the seasons and interactions terms as can be seen from the unstandardized and standardized regression coefficients.

**Table 4:** Becks Anxiety Inventory: regression model with groups and model with seasons

Main Effects	B	SE	LB	UB	β	p	B	SE	LB	UB	β	p
Intercept	3.94	0.40	3.15	4.72		< 0.01*	3.84	0.62	2.63	5.05		< 0.01*

HC (reference)												

MDD	10.92	0.85	9.24	12.60	.46	< 0.01*	11.02	0.86	9.34	12.71	.46	< 0.01*

AAD	11.91	0.85	10.24	13.57	.50	< 0.01*	12.01	0.86	10.33	13.69	.50	< 0.01*

MDD + AAD	-3.93	1.22	-6.33	-1.53	-.16	< 0.01*	-4.05	1.23	-6.47	-1.64	-.16	< 0.01*

Summer (reference)												

Autumn							0.36	0.72	-1.05	1.78	.01	0.61

Winter							-0.54	0.77	-2.05	0.97	.02	0.48

Spring							0.26	0.77	-1.24	1.77	.009	0.73

B = Unstandardized Coefficient

SE = standard error of B

LB = Lower Bound of 95% Confidence Interval for B

UB = Upper Bound of 95% Confidence Interval for B

β = Standardized Coefficient

* p < 0.05

HC = Healthy Control

MDD = Major Depressive Disorder

AAD = Any Anxiety Disorder

MDD + AAD = Major Depressive Disorder + Any Anxiety Disorder

Note: adjusted R^2 ^Model with groups = 0,478

Note: adjusted R^2 ^Model with seasons = 0,477

**Table 5:** Becks Anxiety Inventory: regression model with groups, seasons, covariates and full model with interactions

Main Effects	B	SE	LB	UB	β	p	B	SE	LB	UB	β	p
Intercept	3.09	0.70	1.71	4.46		< 0.01*	2.62	0.72	1.21	4.03		< 0.01*

HC (reference)												

MDD	11.07	0.86	9.39	12.75	.46	< 0.01*	11.81	0.91	10.03	13.60	.49	< 0.01*

AAD	12.02	0.85	10.35	13.70	.50	< 0.01*	11.83	0.85	10.15	13.51	.49	< 0.01*

MDD + AAD	-4.16	1.23	-6.57	-1.76	-.16	< 0.01*	-3.43	1.27	-5.91	-0.95	-.14	< 0.01*

Summer (reference)												

Autumn	0.34	0.72	-1.07	1.75	.01	0.64	0.35	0.72	-1.06	1.76	.01	0.63

Winter	-0.55	0.77	-2.05	0.96	.02	0.48	0.92	1.01	-1.05	2.89	.03	0.36

Spring	0.20	0.77	-1.30	1.71	.007	0.27	1.13	0.88	-0.60	2.86	.04	0.20

Men (reference)												

Women	1.24	0.54	0.17	2.31	.05	0.02*	1.23	0.54	0.16	2.29	.05	0.02*

**Two way interactions**												

Winter & MDD							-2.93	1.28	-5.44	-0.42	-.08	0.02*

Spring & MDD + AAD							-2.86	1.37	-5.53	-0.18	-.06	0.04*

B = Unstandardized Coefficient

SE = standard error of B

LB = Lower Bound of 95% Confidence Interval for B

UB = Upper Bound of 95% Confidence Interval for B

β = Standardized Coefficient

* p < 0.05

HC = Healthy Control

MDD = Major Depressive Disorder

AAD = Any Anxiety Disorder

MDD + AAD = Major Depressive Disorder + Any Anxiety Disorder

Note: adjusted R^2 ^Model with seasons and covariates = 0,480

Note: adjusted R^2 ^Full model with seasons, covariates and interactions = 0,482

### 2.3 Severity of anxiety symptoms (FQ)

**Figure 4:**
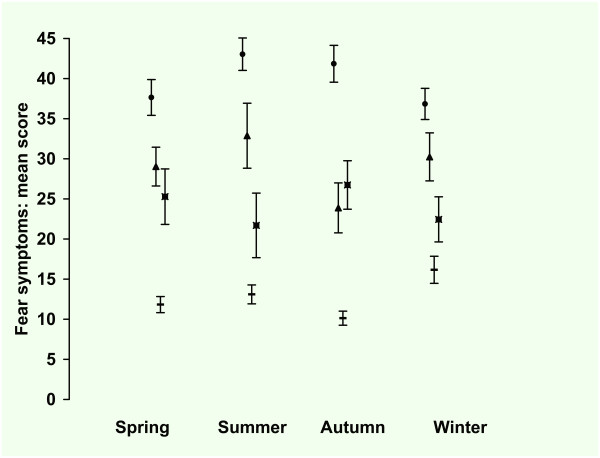
Fear Questionnaire*: mean score per season. * 15 item self report version. Total group (n = 1089), ▬ = Healthy Control (n = 465), ▲ = Any Anxiety disorder last month (n = 133), ■ = Major Depression last month (n = 131), ● = Major Depression and Any Anxiety Disorder last month (n = 360). Values are mean scores. Error bars are Standard Errors of the mean.

The observed mean score was low for autumn (22.9 SE 1.2) and spring (23.9 SE 1.20), and high for summer (26.4 SE 1.35) and winter (27.0 SE 1, 23). The observed mean score for men was 21.9 (SE 0.97) and for women 26.7 (SE 0.81). In Figure 4 the observed means and standard errors of the FQ are presented by season for the four groups.

In tables 6 and 7 the results of the regression analysis are presented. In the model with only groups as predictor, the difference between the groups was significant with a small to large effect size. In the second model with the seasons as predictor there were no significant differences between the seasons. Adding the predictors gender and age revealed that women scored significantly higher than men but there was no significant age effect. In this model with seasons and covariates there was still no significant difference between the seasons. In the full model with seasons, covariates and interactions there were significant two way interactions between season and gender with women scoring higher in summer and autumn compared tot men (+7.1). The difference between women and men was levelled in spring and marginal in winter (+1). There were significant main effects for the groups: patients with MDD scored 12 points higher than HC, patients with AAD scored 17.1 points higher than HC and patients with MDD an AAD scored 27.4 points higher than HC. There was no significant main effect of age and there were no significant two way interactions between age and gender, age and season, age and group, gender and group or season and group. In the final model the effect size was medium for the groups but small for the seasons and interactions terms as can be seen from the unstandardized and standardized regression coefficients.

**Table 6:** Fear Questionnaire: regression model with groups and model with seasons

Main Effects	B	SE	LB	UB	β	p	B	SE	LB	UB	β	p
Intercept	12.29	0.78	10.76	13.83		< 0.01*	13.91	1.21	11.54	16.28		< 0.01*

HC (reference)												

MDD	11.82	1.67	8.55	15.10	.28	< 0.01*	11.81	1.68	8.52	15.10	.28	< 0.01*

AAD	17.02	1.66	13.77	20.28	.41	< 0.01*	16.96	1.67	13.68	20.24	.41	< 0.01*

MDD + AAD	-1.27	2.39	-5.96	3.43	-.03	0.60	-1.23	2.40	-5.94	3.48	-.03	0.61

Summer (reference)												

Autumn							-2.18	1.41	-4.95	0.58	.05	0.12

Winter							-1.62	1.50	-4.57	1.33	.03	0.28

Spring							-2.43	1.50	-5.38	0.51	.05	0.11

B = Unstandardized Coefficient

SE = standard error of B

LB = Lower Bound of 95% Confidence Interval for B

UB = Upper Bound of 95% Confidence Interval for B

β = Standardized Coefficient

* p < 0.05

HC = Healthy Control

MDD = Major Depressive Disorder

AAD = Any Anxiety Disorder

MDD + AAD = Major Depressive Disorder + Any Anxiety Disorder

Note: adjusted R^2 ^Model with groups = 0,335

Note: adjusted R^2 ^Model with seasons = 0,336

**Table 7:** Fear Questionnaire: regression model with groups, seasons, covariates and full model with interactions

Main Effects	B	SE	LB	UB	β	p	B	SE	LB	UB	β	p
Intercept	11.42	1.36	8.75	14.10		< 0.01*	9.56	1.48	1.21	6.65		< 0.01*

HC (reference)												

MDD	11.97	1.67	8.70	15.24	.29	< 0.01*	11.98	1.67	10.03	8.72	.29	< 0.01*

AAD	17.01	1.66	13.75	20.26	.41	< 0.01*	17.07	1.66	10.15	13.82	.41	< 0.01*

MDD + AAD	-1.60	2.39	-6.28	3.09	-.04	0.51	-1.68	2.38	-5.91	-6.36	-.04	< 0.48

Summer (reference)												

Autumn	-2.26	1.40	-5.01	0.49	-.05	0.11	-2.31	1.40	-1.06	-5.05	.05	0.09

Winter	-1.63	1.49	-4.56	1.29	.03	0.27	2.17	2.19	-1.05	-2.13	.04	0.32

Spring	-2.63	1.49	-5.56	0.30	.05	0.78	1.91	2.28	-0.60	-2.56	.04	0.40

Men (reference)												

Women	4.09	1.06	2.01	6.16	.10	< 0.01*	7.10	1.43	0.16	4.29	.17	< 0.01*

**Two way interactions**												

Spring & Women							-7.05	2.60	-5.44	-11.24	-.10	0.02*

Winter & Women							-6.14	2.65	-5.53	-12.25	-.12	< 0.01*

B = Unstandardized Coefficient

SE = standard error of B

LB = Lower Bound of 95% Confidence Interval for B

UB = Upper Bound of 95% Confidence Interval for B

β = Standardized Coefficient

* p < 0.05

HC = Healthy Control

MDD = Major Depressive Disorder

AAD = Any Anxiety Disorder

MDD + AAD = Major Depressive Disorder + Any Anxiety Disorder

Note: adjusted R^2 ^Model with seasons and covariates = 0,344

Note: adjusted R^2 ^Full model with seasons, covariates and interactions = 0,349

### 3.1. Atypical depressive symptoms

**Figure 5:**
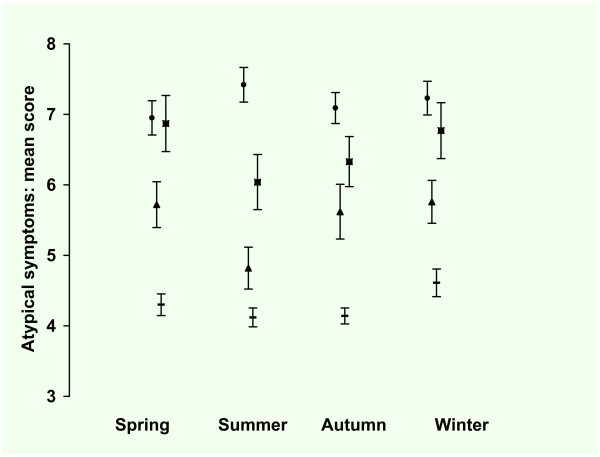
Inventory of Depressive Symptoms*, Atypical symptoms^#^: mean score per season. * 30 Item self-report version of the Inventory of Depressive Symptoms. # Atypical symptoms: summation of the scores on 5 items: mood reactivity, the highest score of either weight gain or increase in appetite, hypersomnia, leaden paralysis, and interpersonal rejection sensitivity (score range 0 - 3, total score range 0 - 15). Total group (n = 1074), ▬ = Healthy Control (n = 446), ▲ = Any Anxiety disorder last month (n = 129), ■ = Major Depression last month (n = 121), ● = Major Depression and Any Anxiety Disorder last month (n = 337). Values are mean scores. Error bars are Standard Errors of the mean.

In Figure 5 the observed means and standard errors of the atypical symptoms are presented by season for the four groups. The observed mean score was lowest for autumn (5.4, SE 0.13) and highest for winter (6.1 SE 0.15), with intermediate scores for spring (5.6 SE 0.14) and summer (5.5 SE .15). The observed mean score for HC was 4.3 (SE 0.07), for patients with AAD 5.5 (SE 0.16), for patients with MDD 6.5 (SE 0.20) and for patients with MDD + AAD 7.2 (SE 0.12). Taking all seasons into account, the observed mean score for men was 5.2 (SE 0.11) and 5.9 (SE 0.09) for women.

In tables 8 and 9 the results of the regression analysis are presented. In the model with only groups as predictor, the difference between the groups was significant with a small to large effect size. In the second model with the seasons as predictor there were no significant differences between the seasons. Adding the predictors gender and age revealed that women scored significantly higher than men and younger participants scored significantly higher than older participants. In this model with seasons and covariates there was no significant difference between the seasons. In the full model with seasons, covariates and interactions there were significant two way interactions between gender and season and between gender and age. Women scored lower in autumn compared to summer (-0.7) and for every additional year of age women scored lower (-0.02), resulting in a 0.9 points diminished score for a 65 year old woman and a 0.5 points diminished score for a 43-year old woman compared to 18-year old woman. There was a significant main effect for the winter compared to summer (+0.3). There were significant main effects for the groups: patients with AAD, MMD and MMD + AAD scored higher than HC (resp. + 1.2, + 2.3, + 2.9). There were no significant two way interactions between age and season, age and group, gender and group or season and group. In the final model the effect size was medium to large for the groups but small for the seasons and interactions terms as can be seen from the unstandardized and standardized regression coefficients.

**Table 8:** Atypical symptoms of the IDS: regression model with groups and model with seasons

Main Effects	B	SE	LB	UB	β	p	B	SE	LB	UB	β	p
Intercept	4.26	0.09	4.08	4.43		< 0.01*	4.16	0.14	3.89	4.43		< 0.01*

HC (reference)												

MDD	2.24	0.19	1.87	2.62	.48	< 0.01*	2.21	0.19	1.83	2.59	.48	< 0.01*

AAD	1.23	0.19	0.86	1.60	.26	< 0.01*	1.19	0.19	0.82	1.57	.26	< 0.01*

MDD + AAD	-0.56	0.27	-1.10	-0.02	-.11	0.04*	-0.51	0.28	-1.10	0.03	-.10	0.06

Summer (reference)												

Autumn							0.02	0.16	-0.30	0.34	.004	0.90

Winter							0.34	0.17	-0.001	0.67	.06	0.51

Spring							0.13	0.17	-0.21	0.47	.02	0.45

IDS = Inventory of Depressive symptoms

B = Unstandardized Coefficient

SE = standard error of B

LB = Lower Bound of 95% Confidence Interval for B

UB = Upper Bound of 95% Confidence Interval for B

β = Standardized Coefficient

* p < 0.05

HC = Healthy Control

MDD = Major Depressive Disorder

AAD = Any Anxiety Disorder

MDD + AAD = Major Depressive Disorder + Any Anxiety Disorder

Note: adjusted R^2 ^Model with groups = 0,312

Note: adjusted R^2 ^Model with seasons = 0,314

**Table 9:** Atypical symptoms of the IDS: regression model with groups, seasons, covariates and full model with interactions

	B	SE	LB	UB	β	p	B	SE	LB	UB	β	p
Intercept	4.27	0.24	3.79	4.74		< 0.01*	3.64	0.34	2.97	4.31		< 0.01*

HC (reference)												

MDD	2.26	0.19	1.88	2.63	.48	< 0.01*	2.25	0.19	1.88	2.62	.48	< 0.01*

AAD	1.19	0.19	0.82	1.56	.26	< 0.01*	1.20	0.19	0.83	1.56	.26	< 0.01*

MDD + AAD	-0.58	0.27	-1.10	-0.05	-.12	0.03*	-0.57	0.27	-1.10	-0.04	-.12	0.04*

Summer (reference)												

Autumn	0.003	0.16	-0.31	0.32	.001	0.98	0.44	0.23	-0.01	0.89	.09	0.54

Winter	0.33	0.17	0.002	0.67	.06	< 0.05*	0.34	0.17	0.01	0.67	.06	< 0.05*

Spring	0.07	0.17	-0.26	0.40	.01	0.67	0.06	0.17	-0.27	0.39	.01	0.73

Men (reference)												

Women	0.65	0.12	0.42	0.89	.14	< 0.01*	1.71	0.40	0.92	2.50	.36	< 0.01*

Age	-0.01	0.004	-0.02	0.03	-.07	< 0.01*	0.00	0.01	-0.01	0.01	.002	0.95

**Two way interactions**												

Autumn & Women							-0.73	0.26	-1.25	-0.22	-.13	< 0.01*

Age & Women							-0.02	0.009	-0.037	-0.002	-.19	0.03*

IDS = Inventory of Depressive Symptoms

B = Unstandardized Coefficient

SE = standard error of B

LB = Lower Bound of 95% Confidence Interval for B

UB = Upper Bound of 95% Confidence Interval for B

β = Standardized Coefficient

* p < 0.05

HC = Healthy Control

MDD = Major Depressive Disorder

AAD = Any Anxiety Disorder

MDD + AAD = Major Depressive Disorder + Any Anxiety Disorder

Note: adjusted R^2 ^Model with seasons and covariates = 0,337

Note: adjusted R^2 ^Full model with seasons, covariates and interactions = 0,344

### 3.2 Melancholic depressive symptoms

**Figure 6:**
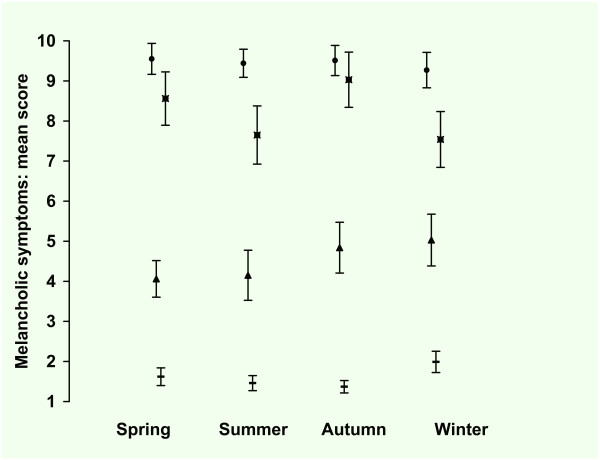
Inventory of Depressive Symptoms*, Melancholic symptoms^#^: mean score per season. * 30 Item self-report version of the Inventory of Depressive Symptoms. # Melancholic symptoms: summation of the scores on the items loss of pleasure, lack of reactivity to usually pleasurable stimuli, depressed mood, regularly worse in the morning, early morning awakening, psychomotor retardation or agitation, the highest score of either anorexia or weight loss, and excessive or inappropriate guilt (score range 0 - 3, total score range 0 - 24). Total group (n = 1033), ▬ = Healthy Control (n = 446), ▲ = Any Anxiety disorder last month (n = 129), ■ = Major Depression last month (n = 121), ● = Major Depression and Any Anxiety Disorder last month (n = 337). Values are mean scores. Error bars are Standard Errors of the mean.

The observed mean score was lowest for summer and autumn (5.0, SE 0.29) and highest for winter (6.0 SE 0.31). The observed mean score for both men and women was 5.3 (SE resp. 0.26 and 0.18). The observed mean score for HC was 1.6 (SE 0.10), for patients with AAD 4.5 (SE 0.30), for patients with MDD 8.2 (SE 0.36) and for patients with MDD + AAD 9.4 (SE 0.20). In Figure 6 the observed means and standard errors of the atypical symptoms are presented by season for the four groups.

In tables 10 and 11 the results of the regression analysis are presented. In the model with only groups as predictor, the difference between the groups was significant with a medium to large effect size. In the second model with the seasons as predictor there were no significant differences between the seasons. Adding the predictors gender and age revealed that there was no significant effect for gender or age. In this model with seasons and covariates there was still no significant difference between the seasons. In the full model with seasons, covariates and interactions there was a significant two way interaction between gender and group: women with a MDD scored lower than men with MDD (-1.1) whereas there was no difference in score between men and women for the other groups. There was also a significant two way interaction between season and group: patients with MDD scored lower in winter (-0.4) compared to the summer whereas the other groups scored higher in winter compared to summer (+0.7). There were significant main effects for the groups: patients with AAD scored higher than HC (+2.9) and patients with MDD + AAD scored 8.8 points higher than HC. Taking the interactions mentioned into account, men with MDD scored higher than HC (+7.5); this was reduced to +6.6 points for women (-0.9) and to +5.5 (-1.1) points for women in winter. There was no significant main effect of age and there were no significant two way interactions between season and gender, age and gender, age and season, age and group. In the final model the effect size was medium to large for the groups but small for the seasons and interactions terms as can be seen from the unstandardized and standardized regression coefficients.

**Table 10:** Melancholic symptoms of the IDS: regression model with groups and model with seasons

Main Effects	B	SE	LB	UB	β	p	B	SE	LB	UB	β	p
Intercept	1.56	0.15	1.28	1.84		< 0.01*	1.42	0.22	0.98	1.86		< 0.01*

HC (reference)												

MDD	6.65	0.31	6.04	7.27	.71	< 0.01*	6.64	0.31	6.03	7.26	.70	< 0.01*

AAD	2.94	0.31	2.34	3.53	.32	< 0.01*	2.94	0.31	2.34	3.54	.31	< 0.01*

MDD + AAD	-1.71	0.45	-2.58	-0.84	-.17	< 0.01*	-1.71	0.45	-2.58	-0.83	-.17	< 0.01*

Summer (reference)												

Autumn							0.19	0.26	-0.33	0.71	.02	0.47

Winter							0.20	0.28	-0.35	0.75	.02	0.48

Spring							0.18	0.28	-0.37	0.72	.02	0.52

IDS = Inventory of Depressive Symptoms

B = Unstandardized Coefficient

SE = standard error of B

LB = Lower Bound of 95% Confidence Interval for B

UB = Upper Bound of 95% Confidence Interval for B

β = Standardized Coefficient

* p < 0.05

HC = Healthy Control

MDD = Major Depressive Disorder

AAD = Any Anxiety Disorder

MDD + AAD = Major Depressive Disorder + Any Anxiety Disorder

Note: adjusted R^2 ^Model with groups = 0,577

Note: adjusted R^2 ^Model with seasons = 0,576

**Table 11:** Melancholic symptoms of the IDS: regression model with groups, seasons, covariates and full model with interactions

Main Effects	B	SE	LB	UB	β	p	B	SE	LB	UB	β	p
Intercept	1.62	0.25	1.12	2.12		< 0.01*	1.28	0.28	0.73	1.82		< 0.01*

HC (reference)												

MDD	6.64	0.31	6.03	7.26	.70	< 0.01*	7.46	0.41	6.64	8.27	.79	< 0.01*

AAD	2.94	0.31	2.34	3.54	.31	< 0.01*	2.89	0.31	2.29	3.50	.32	< 0.01*

MDD + AAD	-1.69	0.45	-2.56	-0.81	-.17	< 0.01*	-1.63	0.45	-2.50	-0.75	-.16	< 0.01*

Summer (reference)												

Autumn	0.20	0.26	-0.31	0.72	.02	0.44	0.23	0.26	-0.28	0.75	.02	0.38

Winter	0.20	0.28	-0.35	0.75	.02	0.47	0.74	0.36	0.03	1.44	.07	0.04*

Spring	0.20	0.28	-0.35	0.74	.02	0.48	0.22	0.28	-0.33	0.76	.02	0.43

Men (reference)												

Women	-0.34	0.20	-0.73	0.05	.04	0.09	0.03	0.26	-0.48	0.55	.003	0.90

**Two way interactions**												

Winter & MDD							-1.06	0.45	-1.95	-0.18	-.07	0.02*

Women & MDD							-0.90	0.40	-1.69	-0.12	-.09	0.02*

IDS = Inventory of Depressive Symptoms

B = Unstandardized Coefficient

SE = standard error of B

LB = Lower Bound of 95% Confidence Interval for B

UB = Upper Bound of 95% Confidence Interval for B

β = Standardized Coefficient

* p < 0.05

HC = Healthy Control

MDD = Major Depressive Disorder

AAD = Any Anxiety Disorder

MDD + AAD = Major Depressive Disorder + Any Anxiety Disorder

Note: adjusted R^2 ^Model with seasons and covariates = 0,577

Note: adjusted R^2 ^Full model with seasons, covariates and interactions = 0,580

## Discussion

The main findings of this study can be summarized as follows. 1) Using a general screening questionnaire, no seasonal pattern in the severity of depressive and anxiety symptoms among primary care patients visiting their general practitioner for any reason could be demonstrated. 2) Among the four diagnostic groups of the NESDA baseline population a small rise of depressive symptoms was found in winter for healthy controls and for patients with any anxiety disorder but neither for patients with a major depression nor for patients with a major depression and an anxiety disorder. Statistical significant differences between the seasons were accompanied by small effect sizes. 3) Both atypical and melancholic symptoms were slightly higher in winter. A distinguishing seasonal pattern in type of (i.e. atypical or melancholic) depressive symptoms could not be demonstrated. For anxiety symptoms (BAI) no seasonal effect could be demonstrated. For symptoms of fear and avoidance (FQ) a small gender related seasonal effect was found with more complaints among women in summer and autumn.

The finding of no seasonal pattern in the severity of depressive and anxiety symptoms in a primary care population (question 1) contrasts to the findings of Mersch et al. [14] who did find seasonality in depressive symptoms as measured with the CES-D scores, and with Oyane et al. [15] who found modest seasonal variations in the depression scores on the Hospital Anxiety and Depression Scale (HADS), both in samples of the general population. These opposing findings might be explained by differences in the populations under scrutiny: general population versus primary care population which could reflect a source of selection bias independent of season. However the results of our study are in line with studies in the general population that failed to demonstrate seasonal fluctuation in the prevalence of depression using general diagnostic instruments like the BDI and the CIDI [10,12]. The results of our study are also supported by Magnusson et al. [11] who reported on the lack of seasonality in anxiety and depression, measured with the HADS, in the Icelandic population and by Blacker et al. [13], who found no significant seasonal variation in General Health Questionnaire scores in a primary care population. A second explanation may be found in the difference in data collection. In the studies by Mersch et al. [14], and Oyane et al. [15] seasonality was assessed retrospectively using questionnaires that assessed fluctuations of symptoms over the year. Like Nayar and Cochrane [25] we think that due to recall bias the retrospective data collection might lead to an overestimation of the seasonal fluctuation in severity and prevalence of affective symptoms. In this study, as in the study by Blacker et al. [13], seasonality was assessed using data on presence and severity of symptoms collected in a cross-sectional way over the year. The cross sectional method of sampling in our study might have introduced a source of selection bias with different groups of patients in different seasons. A third explanation might be that the percentage of patients with a specific seasonal pattern of their complaints in a random population of patients visiting their general practitioner is too small (ranging from 0.4 - 5.6%) to have an effect on the mean scores of depression or anxiety symptoms in that population, even if the severity of their complaints and the health service use of these patients is increased in the winter [26]. For example Blazer et al. [17] found a prevalence of 0,4% of major depression with a seasonal pattern and 1% of major or minor depression with a seasonal pattern in a community based sample, Levitt et al. [27] found a prevalence of 2,9% of seasonal affective disorder in a community sample, Eagles et. al. [6] found a prevalence of 5.3% of seasonal affective disorder during the winter months in a primary care population and Thompson et al. [1] found a prevalence of 5.6% of seasonal affective disorders in a primary care population.

The finding of minimal seasonal differences in severity of depressive or anxiety symptoms (question 2) in various clinical groups is in agreement with the study of Posternak and Zimmerman [9], who did not find higher rates of depressive symptoms in winter in an out-patient population. Seasonal fluctuation of depressive symptoms was even minimized in the group of patients with MDD possibly reflecting the fact that seasonality is usually associated with minor depression, and that once the threshold for a major depression has been passed, seasonality is less influential [3,15,28]. According to the results of this study it can not be ruled out that seasonal mood changes are masked by use of medication or other types of treatment [29,30].

The third finding was that both atypical and melancholic symptoms were slightly more present in winter (question 3). We had expected to find more atypical depressive symptoms in winter, as Blacker [13] did in his study among primary care patients. Literature on seasonal affective disorder describes an atypical symptom profile as a feature of seasonality [3,25,30-32] though others did not find this [33]. An explanation might be the probably low prevalence of seasonal affective disorders in this sample leading to a very small effect on the mean scores of depression or anxiety symptoms in this sample. Another explanation might be an insufficient specificity of the atypical and melancholic specifiers as defined by the DSM IV and used in this article. Baumeister and Parker [34] pointed to the overlap between the different subtyping models of depression. Like Lamers et al. [35] they drew attention tot the ongoing debate on the best criteria to delineate melancholic depression from atypical depression and other depressive conditions. There is considerable discussion whether in atypical depression rejection sensitivity should be included as a main criterion instead of mood reactivity. Novick et al. [36] argued that anxiety is even a more central feature to atypical depression than mood reactivity. In addition to this, they reported that atypical depression shows more co-morbidity with anxiety symptoms than melancholic depression and more likely occurs in younger women.

This study has several strengths: its large sample size and its diagnostic procedures based on standardized structured interviews. Bias caused by participants that were left out of the analysis due to missing items is limited, because those participants represent no more than 1,5% (atypical symptoms) to 5,2% (melancholic symptoms) of the total patient population. The major limitations of this study were its cross-sectional and naturalistic design where seasonal effects may be masked by treatment effects. Another limitation of this study is the lack of specific instruments, such as the SPAQ, to determine whether the previous course of illness had a seasonal pattern. Finally selection bias can not be ruled out completely because data were not primary collected to answer this study assignment.

## Conclusions

Seasonal differences in severity or type of depressive and anxiety symptoms, as measured with a general screening instrument and symptom questionnaires, were absent or small in effectsize in a primary care population and in patient populations with a major depressive disorder and anxiety disorders. For the detection of individuals with a seasonal pattern in depressive episodes more specific questionnaires and a longitudinal approach are needed. These analyses will be forthcoming in our next study on NESDA data.

## Competing interests

The infrastructure for the NESDA study (http://www.nesda.nl) is funded through the Geestkracht program of the Netherlands Organisation for Health Research and Development (ZonMw, grant number 10-000-1002) and is supported by participating universities and mental health care organizations (VU University Medical Center, GGZ inGeest, Arkin, Leiden University Medical Center, GGZ Rivierduinen, University Medical Center Groningen, Lentis, GGZ Friesland, GGZ Drenthe, IQ Healthcare, Netherlands Institute for Health Services Research (NIVEL) and Netherlands Institute of Mental Health and Addiction (Trimbos).

YM has received research funding and served as a consultant for Royal Philips Electronics NV and The Litebook Company Ltd. WAN has received grants from the Netherlands Organization for Health Research and Development, the European Union, the Stanley Medical Research Institute, Astra Zeneca, Eli Lilly, GlaxoSmithKline and Wyeth; has received honoraria/speaker's fees from Astra Zeneca, Pfizer, Servier and Wyeth; and has served in advisory boards for Astra Zeneca, Pfizer and Servier. All authors deny any conflicts of interest or commercial associations in connection with the submitted manuscript.

## Authors' contributions

WHW is PhD candidate and working as a psychiatrist at the University Medical Centre in Groningen. He was involved in all phases of the study and drafted the manuscript. WJP supervised the statistical analysis. YM contributed to the outline of the study and the interpretation of the results. BWHJP, the general principal investigator of the Netherlands Study of Depression and Anxiety (NESDA), approved of the analysis plan. WAN, senior investigator in Groningen for NESDA, supervised all stages of the study. All authors contributed to the manuscript.

## Pre-publication history

The pre-publication history for this paper can be accessed here:

http://www.biomedcentral.com/1471-244X/11/198/prepub
